# Physical exercise intervention in glycogen storage disease IIIa: Feasibility and multisystem benefits

**DOI:** 10.1113/EP092644

**Published:** 2025-05-31

**Authors:** Asunción Bustos‐Sellers, Almudena Montalvo‐Pérez, Araceli Boraita, Gonzalo Saco‐Ledo, Lidia B. Alejo, David Barranco‐Gil, Pedro L. Valenzuela, Nerea López‐Maldonado, Roser Ferrer‐Costa, Javier Esparcia‐Estrela, Celia Monteagudo‐López, Tomàs Pinós, Alejando Santos‐Lozano, Alejandro Lucia, Alfredo Santalla

**Affiliations:** ^1^ Department of Sport and Computer Science, Section of Physical Education and Sports, Faculty of Sport Universidad Pablo de Olavide Sevilla Spain; ^2^ Department of Real Madrid Graduate School, Faculty of Medicine, Health and Sports Universidad Europea de Madrid Madrid Spain; ^3^ Department of Sports Sciences, Faculty of Medicine, Health and Sports Universidad Europea de Madrid Madrid Spain; ^4^ Research Institute of Hospital ‘12 de Octubre’ (‘imas12’) Madrid Spain; ^5^ Health Center CAP Fundación Asistencial Mútua Terrasa Barcelona Spain; ^6^ Spanish Association of Patients With Glycogenosis (AEEG) Barcelona Spain; ^7^ Clinical Biochemistry Department Vall d'Hebron University Hospital Barcelona Spain; ^8^ Clinical Biochemistry, Drug Delivery and Therapy Research Group Vall d'Hebrón Research Institute Barcelona Spain; ^9^ Biomedical Research Networking Center on Rare Disorders (CIBERER) Barcelona Spain; ^10^ Mitochondrial and Neuromuscular Disorders Unit, Vall d'Hebron Institut de Recerca Universitat Autònoma de Barcelona Barcelona Spain; ^11^ i+HeALTH Miguel de Cervantes European University Valladolid Spain; ^12^ Biomedical Research Networking Center on Frailty and Healthy Aging (CIBEFES) Madrid Spain

**Keywords:** aerobic capacity, echocardiograph, glycogenosis, muscle strength, resistance training

## Abstract

Glycogen storage disease III (GSD‐III) is caused by an inherited deficiency of the glycogen debranching enzyme. Affecting the liver, muscle and heart, GSD‐IIIa is the most common GSD‐III subtype. We evaluated the feasibility and safety of a physical exercise intervention in patients with GSD‐IIIa and its effects at the multisystem level. Nine patients (four female; five children and four adults; 60% of all diagnosed Spanish patients) participated in a 12 week home‐based, remotely supervised programme combining moderate‐intensity endurance and resistance exercises. Training tolerance/adaptation (fatigue severity scale, wellbeing questionnaire) and safety were assessed throughout the intervention period. The following outcomes were determined at baseline and postintervention: Health‐related quality of life; muscle, liver, kidney and cardiac damage/function biomarkers; dual X‐ray absorptiometry‐determined fat/muscle/bone mass; echocardiography‐determined cardiac dimensions and left ventricular global longitudinal strain; endurance capacity indicators during cardiopulmonary exercise testing; and muscle strength. The patients completed a median of 88% (interquartile range, 87%–100%) of the prescribed sessions. The intervention was safe and well tolerated, with no changes in wellbeing over time and with a decrease in perceived fatigue severity during the intervention (*p* = 0.002). Barring alanine aminotransferase, there were no changes in blood biomarkers, and the following significant effects were found at postintervention: Decrease in visceral adipose tissue (*p* = 0.038); healthier cardiac geometry [from concentric to normal remodelling, with decreases in relative (*p* = 0.015) and posterior (*p* = 0.042) wall thickness]; and increases in peak oxygen uptake (*p* = 0.033), peak power output (*p* = 0.017) and knee‐extension isometric strength (*p* = 0.012). Although more research is needed, the present findings suggest that exercise should be considered in GSD‐IIIa management.

## Abstract (highlights)

- **What is the central question of this study?**
  What is the feasibility/safety of exercise intervention in patients with glycogen storage disease IIIa and its multisystemic effects?
- **What is the main finding and its importance?**
  In nine affected patients (both sexes, five children), a 12 week exercise (endurance + resistance) programme was safe and well tolerated. The intervention decreased visceral adipose tissue, induced healthier cardiac geometry (from concentric to normal remodelling) and increased peak oxygen uptake, peak power output and knee‐extension isometric strength. Albeit preliminary, these results are clinically promising, because glycogen storage disease IIIa treatment is currently limited to symptom management and diet.

## 1 INTRODUCTION

Glycogen storage disease type III (GSD‐III, also known as Cori disease; OMIM #232400) is a rare metabolic condition caused by an inherited deficiency of the glycogen debranching enzyme, with subsequent accumulation of abnormally structured glycogen (‘limit dextrin’, i.e., the short outer glycogen chains) in different tissues (Hijazi et al., 2021). GSD‐IIIa, which typically affects the liver, muscle and heart tissue, is the most common GSD‐III subtype, accounting for 85% of all cases (Hijazi et al., 2021).

The predominant biochemical features of GSD‐IIIa are hypoglycaemia with/without ketosis, hyperlipidaemia and elevated liver transaminases (Kishnani et al., 2010). Hepatomegaly and hepatic fibrosis can occur at an early age and progress over time (Halaby et al., 2019), with potential long‐term complications being hepatic cirrhosis and hepatocellular adenoma or carcinoma (Demo et al., 2007; Kishnani et al., 2010; Sentner et al., 2016). At the skeletal muscle level, patients with GSD‐IIIa present with exercise intolerance, which is mostly attributable to impaired muscle glycogenolysis (Hennis et al., 2022; Hoogeveen et al., 2021; Preisler et al., 2013; Wary et al., 2010). Thus, patients typically show low levels of muscle strength (Hennis et al., 2022) and cardiorespiratory fitness [peak oxygen uptake (${\dot{V}}_{O_{2}\mathrm{peak}}$)] (Hennis et al., 2022; Preisler et al., 2015, 2013). In contrast, left ventricular LV) hypertrophy (LVH) is the most frequent cardiac manifestation (Vertilus et al., 2010). Although cardiac problems are not always symptomatic (Vertilus et al., 2010), they can have fatal consequences, with some cases reported of heart transplantation in adults (Austin et al., 2012; Cochrane et al., 2007; Hijazi et al., 2021), sudden cardiac death directly (Austin et al., 2012) or at least indirectly associated with cardiopathy (Akazawa et al., 1997; Miller et al., 1972; Shen et al., 1997) or malignant arrhythmias (Tada et al., 1995).

Treatment for GSDIII is currently limited to symptom management and dietary manipulation. Although dietary management traditionally included frequent feeds and/or cornstarch to prevent fasting hypoglycaemia, which is attributable to the impaired ability of the liver to release glycogen‐derived glucose into the bloodstream, this also increases limited dextrin storage and does not slow the progression of cardiac manifestations (Marusic et al., 2020). In fact, the GSDIII consensus guidelines recommend high protein and low complex carbohydrates, with avoidance of simple sugars (Kishnani et al., 2010). To the best of our knowledge, however, no previous study has assessed the effects of another major lifestyle intervention, physical exercise, in the context of GSD‐IIIa. This is an important question given the meta‐analytical evidence showing the beneficial effects (notably, improvements in ${\dot{V}}_{O_{2}\mathrm{peak}}$) of exercise interventions in other GSDs, GSD‐II (Pompe disease) and GSD‐V (McArdle disease) (Bordoli et al., 2022). In this effect, a distinguishing feature of GSD‐IIIa is the multisystemic involvement, versus solely skeletal muscle tissue involvement in GSD‐V (Lucia et al., 2021) or mainly skeletal muscle and cardiovascular (but not liver, at least in adults) involvement in GSD‐II (van Kooten et al., 2021). Additionally, adults with GSD‐IIIa often show more severe cardiac tissue effects (at least in terms of LVH) than those with the late‐onset form of GSD‐II (Boentert et al., 2016; Soliman et al., 2008). Thus, GSD‐IIIa might represent a unique model to evaluate the potential effects of exercise at the multisystem level, including the heart tissue in affected adults.

The main purpose of the present study was to evaluate the feasibility and safety of a home‐based, remotely supervised exercise training programme (combining endurance and resistance exercise) in patients with GSD‐IIIa. A secondary aim was to assess the effects of this type of intervention on health‐related quality of life, blood biomarkers of muscle, liver, kidney and cardiac damage/function, dual X‐ray absorptiometry (DXA)‐determined fat/muscle/bone mass, echocardiography‐determined cardiac dimensions and LV global longitudinal strain, endurance capacity indicators during cardiopulmonary exercise testing and muscle strength.

## 2 MATERIALS AND METHODS

### 2.1 Ethical approval

Written informed consent was obtained from all participants. The study conformed to the standards set by the latest revision of the *Declaration of Helsinki* except for registration in a database, and all the procedures were approved by a properly constituted ethics committee (reference 16081, Instituto de Investigación ‘*imas12*’, Madrid, Spain).

### 2.2 Participants

Eligibility criteria were as follows: female/male genetically diagnosed with GSD‐IIIa and having no major condition contraindicating severe exercise (ischaemic heart disease, uncontrolled hypertension, pericarditis, dilated heart disease or recent acute musculoskeletal disease) or DXA assessment (pregnancy). All the Spanish patients diagnosed with GSD‐IIIa (*n* = 15; 7 children and 8 adults) and their parents (in the case of children) were identified before the start of the study through the Spanish Association of Patients with Glycogenosis (https://www.glucogenosis.org/) and invited to join an online meeting where the possible risks and benefits of the intervention were explained. Eleven patients, all meeting the inclusion criteria, attended the meeting, and they all agreed to participate in the study after signing a consent form.

### 2.3 Intervention

The participants performed a 12 week home‐based combined (endurance and resistance) exercise training programme, in which all the sessions (except the first two, which were directly supervised in person to familiarize and instruct the participants) were remotely and individually supervised by a fitness specialist (A.B.‐S. or A.M.‐P.). All the patients recorded the data of their training sessions in a Google Drive sheet and had weekly online meetings with the relevant fitness specialist.

Endurance training was carried out using a stationary bicycle thrice a week (Monday, Wednesday and Friday). The intensity of all the sessions was controlled with heart rate (HR) monitors (FT 80 W, Polar Electro; Kempele, Finland) and corresponded to 50%–60% of the peak HR obtained in the baseline ramp test that is described further below. The duration of endurance training sessions (excluding warm‐up and cool‐down) increased gradually during the intervention (depending on individual tolerance) from 30 min (start of the programme) to 60 min (end of the programme).

Resistance training was carried out twice a week (Wednesday and Friday) with dumbbells or the participant's own body weight and included two or three sets of six to eight repetitions, with long (2–3 min) rest periods between each set of repetitions and exercises. The following exercises were performed: glute bridge, dead bug, bent‐over dumbbell row knee on bench, dumbbell lunge, isometric quadruped, anti‐rotation core exercise and horizontal chest press. The sessions finished with a structured cool‐down protocol including dynamic muscle stretching and mobility exercises. The intensity was controlled using rate of perceived exertion (RPE) and pain (RPP) 0–10 scales (Hawker et al., 2011). Following the previous methodology used by us in GSD‐V (Santalla et al., 2014), the effort intensity was controlled at the end of each set such that RPE equalled 5–7. When RPE decreased in two consecutive sessions with the same load (in kilograms), the load was increased to achieve again (and maintain) an RPE value of 5–7, while RPP was always ≤1 at the end of each set.

Patients maintained their usual high‐protein diet (Dagli et al., 2009). Both children and adults were administered protein supplementation totalling 3–4 g/kg/day (with regular supervision of kidney function through blood analyses), with mild restriction of carbohydrate supplementation, in the form of cornstarch [*maizena*, i.e., ≤10 g (children) and ≤5 g (adults) in breakfast, lunch, and dinner, respectively, and ≤5–10 g (children) and ≤2.5 g (adults) in mid‐morning and mid‐afternoon snacks]. Participants were also instructed to restrict fat intake to avoid excessive calorie intake. Four‐day dietary intake was assessed at the start and end of the intervention with the Automated Self‐Administered 24‐hour (ASA24) Dietary Assessment Tool; in children, we applied the ASA24 Kids‐2014 (https://epi.grants.cancer.gov/asa24/). This web‐based tool has been shown to be acceptable overall and feasible in adults and children (Kirkpatrick et al., 2017) and to be valid in comparison to true intake in adults (Kirkpatrick et al., 2014) and children (Krehbiel et al., 2017).

### 2.4 Primary outcome (feasibility and safety)

We considered a session completed when the patient reported that 90% of the prescribed exercises were performed successfully, at the planned intensity (Santana Sosa et al., 2012). Tolerance and adaptation to training were monitored daily using a wellbeing questionnaire (McLean et al., 2010), in which fatigue, sleep quality, general muscle soreness, stress level and mood are self‐reported on a five‐point Likert scale. This questionnaire has been widely used in sport training (Buchheit et al., 2013; Gallo et al., 2017; McLean et al., 2010) because it is quick and easy to answer and has shown high between‐day reliability (Thorpe et al., 2017). Muscle stiffness and delayed onset of muscle soreness were also reported using a five‐point scale. Fatigue was measured every 4 weeks (weeks 0, 4, 8 and 12) using the Spanish version of the Fatigue Severity Scale (FSS) (Krupp et al., 1989). The FSS has been used previously in an exercise intervention for adult patients with GSD‐II (Favejee et al., 2015). The patients also reported any potential adverse effects associated with each exercise session.

### 2.5 Secondary outcomes

The secondary outcomes were assessed 2 days before (i.e., baseline) and after the exercise intervention, in the exercise physiology laboratory at the Universidad Europea de Madrid (Spain). Patients reported to our laboratory at 08.00 h, and outcomes were assessed consistently in the same order as reported below. Patients were instructed to refrain from any type of strenuous exercise or physical activity in the 48 h prior to assessments.

#### 2.5.1 Health‐related quality of life

In adult patients, health‐related quality of life was determined using the SF‐36 Health Survey Questionnaire (Spanish version 2.0) (Ware & Sherbourne, 1992), which has been used previously in GSD‐IIIa (Hennis et al., 2022) and GSD‐V (Munguia‐Izquierdo, 2014), whereas in children we used the KIDSCREEN‐27 European Study of Health and Well‐being of Children and Adolescents questionnaire (Spanish version) (The Kidscreen Group, 2006).

#### 2.5.2 Blood biomarkers of muscle, liver, kidney and cardiac damage/function

Patients were told to have a routine blood analysis (in the morning, after overnight fasting) performed in their medical reference centre in the week before both baseline and postintervention examination (the latter ≥24 h after the last resistance training session) to determine the following variables: creatine kinase (CK; as a marker of muscle damage; Banfi et al., 2012; Brancaccio et al., 2010); transaminases (as indicators of liver function); and creatinine (as an indicator of kidney function) through determination of the participants’ estimated glomerular filtration rate using either the chronic kidney disease epidemiology collaborative equation (adults) (Levey et al., 2009) or the Schwart creatine‐based equation (children) (Schwartz et al., 1976; Selistre et al., 2016). Additionally, during both baseline and postintervention visits to our laboratory we obtained blood samples from patients by venipuncture to determine, using immunoassay kits (Atellica Solution, Siemens Healthineers, Madrid, Spain), the serum levels of the following two cardiac biomarkers in resting conditions and upon completion of the cardiopulmonary exercise tests that are described below: N‐terminal prohormone of brain natriuretic peptide (NT‐proBNP, a molecule secreted by cardiomyocytes in response to increased wall stress, i.e., a blood biomarker for heart failure severity and subclinical LV dysfunction; Welsh et al., 2022); and high‐sensitivity cardiac troponin‐I (hs‐cTnI), which allows the detection of considerably low concentrations of cardiac troponin and can be used as a predictor of cardiovascular events and mortality in apparently healthy individuals (Farmakis et al., 2024).

#### 2.5.3 Body composition

Body height and mass were assessed with a stadiometer (Seca 711; Hamburg, Germany) and a balanced scale (Ano Sayol SL; Barcelona, Spain), respectively, with the participants in their underwear. Body composition was assessed by DXA (Hologic Serie Discovery QDR, Software Physician's Viewer, APEX System Software version 3.1.2.; Bedford, MA, USA), as explained elsewhere (Santalla et al., 2022). We determined body fat [including estimated visceral adipose tissue (VAT)], lean mass and bone mineral content/density.

#### 2.5.4 Echocardiography

Resting cardiac dimensions were assessed by an experienced cardiologist (A.B., >35 years of experience). All procedures were performed using an EPIQ CVx system (Philips; Amsterdam, The Netherlands) equipped with a colour tissue Doppler, multifrequency 2.4 MHz transducer. Interventricular septum (IVS) thickness, LV posterior wall (LVPW) thickness and LV end‐diastolic diameter (LVEDD), were measured using two‐dimensional guided M‐mode imaging following recommendations from the American Society of Echocardiography (Lang et al., 2015), with participants in a supine position. We also calculated LV end‐diastolic volume, LV mass, LV ejection fraction (LVEF) and LV fractional shortening (LVFS) as determined elsewhere (Boraita et al., 2022), in addition to relative wall thickness (RWT) using the following formula: RWT = (IVS + LVPW)/LVEDD (Boraita et al., 2022).

In children, LVH was considered using the age‐specific >95th percentile for LV mass indexed by height (in grams per metre to the power of −2.7) presented in the reference data from Khoury et al. (2009) and classified as concentric or eccentric (if present together with RWT of ≥0.42 and  0.42, respectively) (Daniels et al., 1998). In the absence of LVH, LV geometry remodelling was defined as normal (RWT of <0.42) or concentric (RWT ≥ 0.42) (Daniels et al., 1998). In adults, LVH was considered if LV mass was >116 g/m^2^ (males) or >96 g/m^2^ (females) and classified as concentric or eccentric (if present together with RWT of ≥0.42 or <0.42, respectively); in the absence of LVH, LV geometry remodelling was defined as normal (RWT of <0.42) or concentric (RWT of ≥0.42) (Lang et al., 2015).

Additionally, two‐dimensional speckle tracking echocardiography was used to determine LV global longitudinal strain (GLS) with participants resting supine, as recently reported for patients with GSD‐V (Santos‐Lozano et al., 2024). The Auto Strain function, within the Philips EPIQ CVx ultrasound system, was used for out‐of‐cart strain assessment on saved records. GLS values were obtained by analysing multi‐camera perspectives, including two‐, three‐ and four‐camera views. The software provides longitudinal strain curves, and the GLS was calculated subsequently by averaging the values obtained in all segments of the myocardium. A second cardiologist reviewed the Auto Strain analysis to confirm the accuracy of the obtained GLS values or, if necessary, to adjust manually any segments where the automated tracking showed inconsistencies or artefacts.

#### 2.5.5 Cardiopulmonary exercise tests

All the tests were performed in similar environmental conditions (20°C–24°C, 45%–55% relative humidity) and at the same time of day (from 10.00 to 12.00–13.00 h) using an electromechanical brake cycle ergometer (800s, Ergoline, Bitz, Germany), at a pedal cadence of 60–80 r.p.m., and with gas‐exchange data continuously recorded (CPX Ultima, Medical Graphics Corporation, St Paul, MN, USA). Participants initially performed a 15 min constant‐load moderate‐intensity bout, in which the workload was increased progressively until they reached 60% of their age‐estimated maximum HR (220 minus age, in years) in the first 3 min, and this workload was maintained for a total of 15 min (Valenzuela, Santalla, Alejo, Bustos et al., 2024; Valenzuela, Santalla, Alejo, Merlo et al., 2024). Immediately thereafter, they performed a ramp test until exhaustion. This test started at the workload applied in the constant bout, which was subsequently increased by 5 W (children) or 10 W (adults) every 60 s until volitional exhaustion or when one of the following criteria was met: contracture (or near‐start of contracture) or inability to keep a pedal cadence ≥60 r.p.m. (Valenzuela, Santalla, Alejo, Bustos et al., 2024; Valenzuela, Santalla, Alejo, Merlo et al., 2024).

At the end of the constant‐load bout (average of the last 1 min), substrate (fat and carbohydrate) oxidation was estimated with the equations proposed elsewhere (Jeukendrup & Wallis, 2005). Additionally, the workload eliciting the ventilatory threshold (VT) was identified visually by two independent investigators (A.S. and L.B.‐A.) or by a third one (D.B.‐G.) in the case of disagreement as the workload eliciting an increase in the 20 s average value of the ventilatory equivalent for oxygen but with no concomitant increase in the ventilatory equivalent for carbon dioxide (Mate‐Munoz et al., 2007). The ${\dot{V}}_{O_{2}\mathrm{peak}}$ was determined as the highest O_2_ uptake value (20 s average) recorded during the ramp tests (Valenzuela, Santalla, Alejo, Merlo et al., 2024).

Blood lactate (Lactate Plus, Nova Biomedical, Waltham, MA, USA), glucose (FreeStyle Freedom Lite, Abbott, Chicago, IL, USA) and ketone levels (GKI, Keto‐Mojo, Emeryville, CA, USA) were assessed in capillary blood samples obtained from fingertips at baseline and upon completion of the constant‐load and ramp tests, respectively.

#### 2.5.6 Muscle strength

The maximum isometric strength of participants was assessed using a dynamometer (KForce, Kinvent, Montpellier, France) (Pienaar & Barnard, 2017). Lower‐limb (knee‐extension exercise, in a prone position with knee flexion at 90°) and upper‐limb (chest press exercise, in supine position with elbow flexion at 90°) isometric muscle strength was determined. Three measurements of 3 s duration (interspersed by a 2 min recovery) were performed on each side (always starting on the left side), and the maximum force (in kilograms) was determined as the highest value reached (and as the average of the maximum value for left and right lower limb in knee‐extension exercise).

### 2.6 Statistical analysis

Data are shown as the median and interquartile range. Differences between baseline and postintervention outcomes were assessed with the Wilcoxon signed‐rank test. The effect size was calculated using the rank‐biserial correlation (*r*), derived from the *Z*‐value of the Wilcoxon signed‐rank test, following the formula *r* = *Z*/√*N*, where *N* is the total number of observations. For those outcomes that were measured more than twice over time (adaptation to training, blood response to exercise tests) the Friedman test was performed, and the effect size was calculated using 𝜂^2^, based on the Friedman χ^2^ statistic and the formula *η* ^2^ = χ^2^/[*n*(*k* − 1)]. Additionally, we calculated the non‐parametric Spearman's rank correlation coefficient (ρ) to determine the correlation between adherence to the exercise intervention (i.e., completion rate), on the one hand, and the significant intervention‐induced changes, on the other. Statistical analyses were conducted using a statistical software package (SPSS v.23.0; IBM Corp., Armonk, NY, USA). The level of statistical significance was set at 0.05.

## 3 RESULTS

One adult patient decided, after undergoing the baseline laboratory assessments, not to start the intervention. One child left the study during the first weeks for reasons not related to the intervention. Thus, four adults (one female) and five children (three females) completed the intervention, and their data were included in the analyses (Table 1). There were no differences in 4‐day dietary intake at baseline versus postintervention, except for an increase and decrease in the percentage of protein and fat, respectively, in children (Supplemental File 1).

**TABLE 1:** Individual descriptive characteristics of the participants at the start of the study.

Patient number	Age (years)	Weight (kg)	Height (cm)	Pathogenic mutation in the two *AGL* alleles	
1 (male)	10	43.2	142.0	c.3216_3217delGA	c.3444C>A
2 (female)	12	43.1	152.0	c.3971_3972delAT	p.yr1324Term
3 (female)	9	40.6	143.5	c.3216_3217delGA	c.2711_2717delins13
4 (female)	14	43.4	148.0	c.1423+1G>A	c.2681+1G>A
5 (male)	14	43.6	160.0	c.4260‐12ª>G	c.4260‐12ª>G
6 (male)	45	66.2	172.5	c.3216_3217delGA (p.Glu1072AspfsTer36)	c.3216_3217delGA (p.Glu1072AspfsTer36)
7 (male)	35	78.6	178.0	c.3980G>A	c.348_373del26
8 (female)	33	77.8	156.5	p.glu1072AspFs*36	pAla117LeuFs*10
9 (male)	42	67.6	173.0	c.3103_3104insA	c2390A>G

Abbreviation: *AGL*, glycogen debranching enzyme gene.

### 3.1 Primary outcomes (feasibility and safety)

The patients successfully completed a median of 88% of the prescribed training sessions (interquartile range, 87%–100%), which corresponded to 88% (interquartile range, 87%–100%) and 91% (interquartile range, 79%–95%) for children and adults, respectively. The intervention seemed to be well tolerated, in that there were no changes in wellbeing over time and the patients reported a significant decrease in perceived fatigue severity during the intervention (*p* = 0.002; Table 2). Additionally, there were no major adverse events during or after any of the sessions. Three patients reported muscle soreness on some days (associated with the lunge exercise), and one girl required a load readjustment in the progression of resistance loads (i.e., she reported RPP values of >1 during a session, and the load was reduced by two to four repetitions per set in the next session to ensure that RPP values were consistently ≤1).

**TABLE 2:** Tolerance and adaptation to the intervention: Fatigue and wellbeing.

	Week 0	Week 4	Week 8	Week 12	*p*‐value (effect size^a^)
FSS (*n* = 8)	6.0 (5.1–6.4)	4.0 (3.4–5.7)	3.2 (2.7–5.1)	2.5 (2.2–3.8)	**0.002** (0.609)
Wellbeing questionnaire^b^					
Fatigue	3.3 (3.3–4.3)	3.8 (3.0–4.4)	3.7 (2.9–4.0)	3.7 (3.0–4.0)	0.451 (0.098)
Sleep quality	4.0 (4.0–4.2)	4.0 (4.0–4.5)	4.0 (3.9–4.3)	4.2 (4.0–4.3)	0.531 (0.082)
General muscle soreness	4.4 (3.9–4.9)	4.8 (4.2–5.0)	4.0 (3.5–4.6)	4.3 (3.5–4.8)	0.357 (0.120)
Stress level	4.0 (3.6–4.1)	3.9 (3.7–4.0)	3.3 (3.0–4.0)	3.8 (3.4–4.0)	0.410 (0.107)
Mood	4.0 (4.0–4.0)	4.0 (4.0–4.0)	4.0 (4.0–4.0)	4.0 (4.0–4.0)	0.784 (0.040)
Muscular stiffness	1.0 (0.6–1.3)	0.6 (0.0–0.09)	1.0 (0.3–1.3)	0.6 (0.0–0.9)	0.315 (0.131)
DOMS	0.9 (0.6–1.6)	0.7 (0.0–1.0)	1.0 (0.0–1.3)	1.0 (0.0–1.0)	0.669 (0.058)

*Note*: Data are the median (interquartile range), and the sample size was *n* = 9 for all outcomes except for FSS. The significant *p*‐value is in bold.

Abbreviations: DOMS, delayed onset of muscle soreness; FSS, fatigue severity scale.

^a^ The effect size was calculated using 𝜂^2^, based on the Friedman χ^2^ statistic.

^b^ This questionnaire was administered daily, but only median (interquartile range) values for the relevant weeks are provided for the sake of simplicity.

### 3.2 Secondary outcomes

#### 3.2.1 Health‐related quality of life

No significant intervention effects were found in health‐related quality of life domains in children or adults (with *p* = 0.083 for the domain ‘general health’ in adults; Table 3).

**TABLE 3:** Results of health‐related quality of life.

	Baseline	Postintervention	*p*‐value (effect size^a^)
Adults (SF‐36)			
Physical function	20.0 (18.8–26.3)	27.5 (15.0–38.1)	0.715 (0.183)
Physical role	93.8 (71.9–100)	68.8 (46.9–90.6)	0.655 (0.224)
Bodily pain	51.5 (48.5–54.0)	62.0 (46.8–79.0)	0.465 (0.365)
General health	41.0 (25.0–52.0)	46.0 (30.0–54.5)	0.564 (0.289)
Vitality	53.1 (42.2–70.3)	59.4 (37.5–84.4)	1.000 (0.000)
Social functioning	87.5 (65.6–100)	87.5 (81.3–90.6)	0.593 (0.268)
Emotional role	91.7 (83.3–100)	100 (93.8–100)	0.655 (0.224)
Mental health	75.0 (63.8–80.0)	82.5 (70.0–86.3)	0.414 (0.408)
Children (KIDSCREEN)			
Physical wellbeing	16.0 (16.0–20.0)	20.0 (17.0–20.0)	0.414 (0.365)
Psychological wellbeing	22.0 (21.0–23.0)	23.0 (23.0–23.0)	0.197 (0.577)
Autonomy and parent relationship	32.0 (26.0–32.0)	33.0 (32.0–34.0)	0.144 (0.653)
Social support and peers	18.0 (14.0–19.0)	15.0 (15.0–18.0)	0.336 (0.430)
School environment	18.0 (16.0–19.0)	17.0 (16.0–17.0)	0.276 (0.487)

*Note*: Data are the median (interquartile range), and the sample size was *n* = 4 (adults) and *n* = 5 (children) for all outcomes.

^a^ The effect size was calculated using the rank‐biserial correlation.

#### 3.2.2 Blood biomarkers

All the patients brought the reports of baseline and postintervention blood analyses in the two visits to our laboratory, although some laboratory reports did not include both baseline and postintervention results (i.e., allowing us to perform statistical comparisons) of CK (*n* = 3), alanine aminotransferase (*n* = 2), aspartate aminotransferase (*n* = 2) or gamma‐glutamyl transferase (*n* = 3). In turn, two patients (one child) did not want/consent to have blood samples taken by us before and upon completion of the exercise tests in our laboratory (for hs‐cTnI and NT‐ProBNP determination), and another patient declined blood sampling postexercise.

Median levels of muscle, liver, kidney and cardiac biomarkers did not change with the intervention, except for an increase in alanine aminotransferase (Table 4). The median values of NT‐proBNP were below the limits suggestive of heart failure in the non‐acute setting (i.e., 125 pg/mL; McDonagh et al., 2021), and especially below those indicative of chronic heart failure [i.e., 400 pg/mL; National Guideline Centre (UK), 2018] in both baseline and postintervention assessments. These levels did not change after intense acute exercise (cardiopulmonary exercise testing) in baseline or postintervention assessments. Similar results were obtained for hs‐cTnI.

**TABLE 4:** Blood biomarkers (muscle, liver, kidney and cardiac function).

	Baseline	Postintervention	*p*‐value (effect size^a^)
Liver			
Aspartate aminotransferase, U/L (*n* = 7)	127.0 (86.5–173.5)	141.0 (96.0–182.0)	0.075 (0.673)
Alanine aminotransferase, U/L (*n* = 7)	151.0 (100.5–165.5)	122.0 (97.5–163.0)	**0.034** (0.801)
Gamma‐glutamyl transferase, U/L (*n* = 6)	26.5 (19.0–37.0)	26.0 (18.3–36.8)	0.416 (0.332)
Muscle			
Creatine kinase, U/L (*n* = 6)	2370 (1661–4125)	3224 (2473–3615)	0.173 (0.557)
Kidney			

Creatinine, mg/dL (*n* = 9)	0.57 (0.53–0.64)	0.57 (0.53–0.64)	0.889 (0.047)
eGFR, mL/min/1.73 m^2^ (*n* = 9)	128 (122–175)	132 (125–151)	0.484 (0.233)
Heart			
NT‐ProBNP at rest, pg/mL (*n* = 7)	80 (50–128)	70 (61–77)	0.397 (0.320)
NT‐ProBNP, ∆ (%) with acute exercise (*n* = 6)	14 (5–20)	11 (6–14)	0.345 (0.385)
hs‐cTnI at rest, ng/mL (*n* = 7)	19 (16–57)	14 (12–59)	0.917 (0.040)
hs‐cTnI, ∆ (%) with acute exercise (*n* = 6)	4 (−5 to 4)	−3 (−10 to 9)	0.600 (0.214)

*Note*: Data are the median (interquartile range). The sample size (*n*) available for each measure is shown. The significant *p*‐value is in bold.

Abbreviations: eGFR, estimated glomerular filtration rate; hs‐cTnI, high‐sensitivity cardiac troponin I; NT‐proBNP, N‐terminal prohormone of brain natriuretic peptide.

^a^ The effect size was calculated using the rank‐biserial correlation.

#### 3.2.3 Body composition

No significant intervention effects were noted on body weight or DXA‐determined variables, except for a decrease in VAT at postintervention in visceral adipose tissue (Table 5).

**TABLE 5:** Results of dual energy X‐ray absorptiometry‐determined variables.

Parameter	Baseline	Postintervention	*p*‐value (effect size^a^)
Body mass, kg	43.6 (43.2–67.6)	45.7 (44.2–70.0)	0.109 (0.534)
Fat mass, kg	11.3 (11.2–18.6)	13.0 (11.9–20.2)	0.110 (0.533)
VAT, g	247 (215–414)	273 (205–393)	**0.038** (0.691)
BMD, g/cm^2^	0.97 (0.89–1.03)	0.97 (0.91–1.04)	0.477 (0.237)
BMC, kg	1.5 (1.1–2.1)	1.5 (1.2–2.1)	0.374 (0.296)
Fat‐free mass, total	33.6 (30.8–46.9)	35.1 (30.8–47.7)	0.374 (0.296)

*Note*: Data are the median (interquartile range), and the sample size was *n* = 9 for all outcomes. The significant *p*‐value is in bold.

Abbreviations: BMC, bone mineral content; BMD, bone mineral density; VAT, visceral adipose tissue.

^a^ The effect size was calculated using the rank‐biserial correlation.

#### 3.2.4 Echocardiography

Four of the five children showed concentric cardiac remodelling at baseline, and only one showed normal remodelling. Yet, at postintervention, three of these four children with previous concentric remodelling had shifted to normal remodelling (with the remaining two children maintaining their previous condition). In adults, three patients and one patient showed a normal and eccentric pattern, respectively, at baseline. All of them retained the same pattern at postintervention. None of the patients (children or adults) met the criteria of LVH.

The intervention exerted a ‘thinning’ effect on LV walls, with a significant decrease in RWT (*p* = 0.018) and PWT (*p* = 0.038) (Table 6). No intervention effect was noted for GLS.

**TABLE 6:** Results of echocardiography assessments.

**Parameters**	**Baseline**	**Postintervention**	***p*‐value (effect size** ^a^)
Dimensions			
PWT, mm	8.0 (7.7–9.1)	7.5 (7.1–8.0)	**0.038** (0.691)
SWT, mm	8.4 (7.6–9.3)	7.6 (7.4–9.0)	0.139 (0.494)
LVEDD, mm	40.3 (37.5–48.9)	42.6 (38.6–50.8)	0.441 (0.257)
RWT	0.4 (0.4–0.4)	0.4 (0.3–0.4)	**0.018** (0.792)
LV mass, g/m^2^	73 (65–77)	69 (57–77)	0.137 (0.495)
LVEDV, mL/m^2^	50 (45–62)	53 (47–67)	0.575 (0.187)
Function			
GLS, %	−18.2 (−20.0 to −16.3)	−18.4 (−18.3 to −15.0)	0.123 (0.514)

*Note*: Data are the median (interquartile range), and the sample size was *n *= 9 for all outcomes. Significant *p*‐values are in bold.

Abbreviations: GLS, global longitudinal strain (left ventricle); LV, left ventricle; LVEDD, left ventricular end‐diastolic diameter; LVEDV, left ventricular end‐diastolic volume; PWT, LV posterior wall thickness (diastole); RWT, relative wall thickness; SWT, septal wall thickness.

^a^ The effect size was calculated using the rank‐biserial correlation.

#### 3.2.5 Cardiopulmonary exercise tests

No significant time effect was found for lactate, glucose or ketones (Supplemental File 2). In contrast, the intervention resulted in significant improvements in ${\dot{V}}_{O_{2}\mathrm{peak}}$ and peak power output (Table 7).

**TABLE 7:** Results of physiological responses to exercise.

	Baseline	Postintervention	*p*‐value (effect size^a^)
Constant load test			
Fat oxidation, g/min	0.2 (0.2–0.3)	0.2 (0.2–0.3)	0.171 (0.456)
Carbohydrate oxidation, g/min	0.5 (0.5–0.7)	0.5 (0.4–0.5)	0.161 (0.467)
Ramp test			
VT, W	20 (12–45)	30 (22–45)	0.201 (0.426)
${\dot{V}}_{O_{2}\mathrm{peak}}$, mL/kg/min	17.3 (14.4–19.5)	20.2 (20.0–21.2)	**0.038** (0.691)
Peak power output, W	55 (27–70)	55 (42–95)	**0.017** (0.798)

*Note*: Data are the median (interquartile range), and the sample size was *n* = 9 for all outcomes. Significant *p*‐values are in bold.

Abbreviations: ${\dot{V}}_{O_{2}\mathrm{peak}}$, peak oxygen uptake; VT, ventilatory threshold.

^a^ The effect size was calculated using the rank‐biserial correlation.

#### 3.2.6 Muscle strength

The exercise intervention led to significant improvements in lower‐body (but not in upper‐body) isometric muscle strength (Table 8).

**TABLE 8:** Results of maximum isometric strength.

	Baseline	Postintervention	*p*‐value (effect size^a^)
Chest press			
Maximum force, kg	22.6 (15.7–29.5)	25.6 (18.2–29.6)	0.123 (0.545)
Knee extension (average of both lower limbs)			
Maximum force, kg	22.1 (14.7–25.9)	39.4 (26.6–42.5)	**0.012** (0.891)

*Note*: Data are the median (interquartile range), and the sample size was *n* = 8 for all outcomes (because the dynamometer was temporarily unavailable during the postintervention assessment of one child). The significant *p*‐value is in bold.

^a^ The effect size was calculated using the rank‐biserial correlation.

#### 3.2.7 Correlations between adherence to the intervention and exercise‐induced improvements

No significant correlations were found between adherence to the intervention, on the one hand, and significant intervention‐induced changes (absolute ρ‐values ranging from 0.072 to 0.718, and *p*‐values ranging from 0.074 to 0.913), on the other, except for a positive correlation with respect to an increase in fatigue from week 8 to 12 (ρ = 0.747, *p* = 0.033).

## 4 DISCUSSION

The main new findings of the present study were that a 12 week combined (resistance and endurance) remotely supervised, home‐based, exercise training programme (which was combined with a high‐protein diet) was safe and well tolerated by patients (adults and children) with GSD‐IIIa, while increasing maximum cardiorespiratory fitness (${\dot{V}}_{O_{2}\mathrm{peak}}$), peak exercise capacity and lower‐limb muscle strength. Moreover, the intervention induced an overall beneficial cardiac remodelling pattern, especially in children (i.e., from concentric to normal remodelling). An additional positive effect was a decrease in VAT. Compliance with the intervention was high (i.e., median of 88%), which is similar to previously reported values for exercise interventions of the same duration in adults with GSD‐II [e.g., 32 of 36 planned sessions (89%) for a 12 week combined exercise (endurance, strength and core modalities) and diet intervention in adults with GSD‐II; Favejee et al., 2015].

The results of the fatigue tests provided interesting information. Although the baseline FSS results (median of 6.0) indicated ‘severe fatigue’ (i.e., >4) (Krupp et al., 1989), there was a significant reduction to ‘moderate fatigue’ after the intervention (i.e., median of 2.5, which is close to the average value of 2.3 reported in healthy people; Hewlett et al., 2011). Given that most items of the weekly FSS estimate fatigue in exercise and considering that endurance and resistance training loads were increased during the intervention (being notably higher in the last week than at the start of the intervention), these data suggest a good tolerance to the applied training programme and a notable effectiveness of this intervention to reduce patients’ fatigue. Additionally, although there was a significant increase in a liver transaminase that is known to be released from damaged skeletal muscle tissues (i.e., alanine aminotransferase, *p* = 0.034) (Banfi et al., 2012), the liver‐specific biomarker (indicative of eventual dysfunction of this tissue), gamma‐glutamyl transferase, was unchanged by the intervention (*p* = 0.416) (Whitfield, 2001). Although more research is needed, we believe the results of gamma‐glutamyl transferase further support the overall safety of our intervention.

Our findings showed an average ${\dot{V}}_{O_{2}\mathrm{peak}}$ improvement of ∼15% in the study participants. Although there is no previous evidence on effects of exercise intervention in patients with GSD‐IIIa, our results are in line with meta‐analytical evidence on GSD‐II (Pompe disease; data compiled from *n* = 29 adult patients) and GSD‐V (McArdle disease; *n* = 34 adult patients): a combination of endurance (30 min of cycling) and strength (using either body weight, weights or exercise machines) training three times a week for 12–20 weeks improved ${\dot{V}}_{O_{2}\mathrm{peak}}$ by 9%–10% in patients with GSD‐II, whereas endurance exercise training (three to five weekly sessions during 4–32 weeks, and protocols including ≤60 min of walking, jogging or cycling at moderate intensities, i.e., 60%–85% of peak HR) increased ${\dot{V}}_{O_{2}\mathrm{peak}}$ by 14%–111% in those with GSD‐V (Bordoli et al., 2022). These are important findings given that ${\dot{V}}_{O_{2}\mathrm{peak}}$ (also known as ‘cardiorespiratory fitness’) is a robust, independent prognostic factor of morbidity and mortality from all causes, particularly cardiometabolic conditions (Fletcher et al., 2018; Lavie et al., 2019), even beyond some well‐established risk factors, such as high blood pressure, type 2 diabetes, obesity or smoking (Kavanagh et al., 2003; Kokkinos et al., 2008; Myers et al., 2002). In fact, the American Heart Association advocates for the routine assessment of this measure as a clinical vital sign (Ross et al., 2016). An additional cardiometabolic benefit of the intervention was the reduction (by ∼9%) of estimated VAT (inside the abdominal cavity). In this regard, owing to the close proximity to several organs (particularly the liver, pancreas and heart), excess VAT might adversely affect the metabolic function of these organs, leading to insulin resistance (Valenzuela, Carrera‐Bastos et al., 2023). Furthermore, VAT might have unique properties that are linked to cardiometabolic risk, including the secretion of pro‐inflammatory molecules that spill over into the circulation, leading to a state of systemic chronic inflammation (Valenzuela, Carrera‐Bastos et al., 2023).

The intervention induced beneficial effects at the cardiac tissue level, with no increases in biomarkers of cardiac dysfunction (NT‐ProBNP) or damage (hs‐cTnI). At postintervention, three of the four children with previous concentric remodelling had shifted to normal remodelling, and exercise intervention exerted a ‘thinning’ effect on LV walls, with a significant decrease in RWT. These are findings of potential clinical relevance when considering the high prevalence of cardiac alterations in GSD‐IIIa (including children) and the potential deleterious (and sometimes fatal) progression of potential cardiac manifestations. Indeed, a cardiac condition has been reported by 66% of 29 adults and 46% of 46 caregivers of affected children in a recent survey of patients with GSD‐III from nine countries (Evins et al., 2024). However, none of the participants in our study met the criteria of LVH, which is the most prevalent cardiac manifestation in these patients (although it can be asymptomatic with preserved systolic function, as assessed with LVEF; Vertilus et al., 2010). The incidence of LVH in patients with GSD‐IIIa might increase with age, being reported in three of four adult males and two of seven adult females (Vertilus et al., 2010), in 50% of eight young adults (21–28 years old) (Carvalho et al., 1993) or in 42.9% of 21 adults (19–68 years old) of both sexes (14 female) (Hijazi et al., 2021). Of note, cardiac manifestations can have fatal consequences in GSD‐IIIa. Two of 21 adults were treated with heart transplantation at the ages of 24 and 40 (Hijazi et al., 2021). Cardiac transplantation has also been described in a 27‐year‐old female (Austin et al., 2012) and a 39‐year‐old male (together with liver and kidney transplants) (Cochrane et al., 2007), both after suffering heart failure, and some cases of sudden cardiac death have been reported to be associated, at least indirectly, with GSD‐IIIa (Akazawa et al., 1997; Austin et al., 2012; Miller et al., 1972; Shen et al., 1997). Post‐mortem heart biopsies from three patients revealed moderate (or mild‐to‐moderate) vacuolation (attributable to cytosolic limit dextrin accumulation) in the myocytes of both ventricles and of the right atrium, and in specialized myocytes of the sinoatrial/atrioventricular nodes and in the cardiac conduction system (Austin et al., 2012). An additional novelty of our study was the assessment of GLS in patients with GSD‐IIIa. Indeed, GLS (or deformation of the LV) is considered a good proxy for myocardial contractility of the LV and allows identification of subtle disturbances in systolic function (Karlsen et al., 2019; Potter & Marwick, 2018). The GLS values of our patients ranged within reference levels (i.e., −24% to −16%; Nyberg et al., 2023) and did not increase with the intervention, perhaps suggesting that longer and/or more intense exercise programmes are needed to improve this important indicator. Our results on GLS overall are in accord with recent findings in eight (three female) adult patients with GSD‐V, in whom baseline GLS values averaged ∼−17% and were similar to those of healthy age‐ and sex‐matched control subjects (Santos‐Lozano et al., 2024).

The training intervention increased the maximal isometric strength of the lower limbs, which provides new evidence (although, ideally, controlled trials should be performed) that patients with GSD‐IIIa can perform resistance training and adapt to it. This is clinically relevant when considering not only the natural course of the disease in some patients (whose muscle deterioration can become severe, eventually leading to a loss of the ability to walk, and therefore requiring the use of a wheelchair; Sentner et al., 2016), but also the fact that the World Health Organization recommends that physical muscle‐strengthening activities should be performed regularly by essentially all population groups (Bull et al., 2020). Indeed, muscle strength is gaining importance as a health indicator in general, including cardiovascular‐associated mortality (Valenzuela, Ruilope et al., 2023). Based on our previous experience with resistance training in GSD‐V (Santalla et al., 2014), the low number of repetitions being used during the sessions allowed the use of muscle phosphocreatine as the main energy substrate to fuel muscle contractions, with no major reliance on glycogen deposits; and the training structure, with long (2–3 min) rest periods between each set of repetitions and exercises was designed to allow phosphocreatine to be resynthesized in a given muscle before this muscle was used again. However, our remotely supervised, home‐based programme was unable to increase the total muscle mass of the patients. This is apparently in contrast to a previous report in patients with GSD‐V, in which a 4 month training intervention focusing solely on resistance exercises, in which all the sessions were individually supervised (in person), significantly increased total muscle mass (by +855 g on average; Santalla et al., 2014). In contrast, a 2 year home‐based combined (endurance and resistance) programme failed to induce significant improvements in the muscle mass of patients with GSD‐V (Santalla et al., 2022). It therefore seems that individual supervision in person is needed for resistance training sessions to induce a significant gain in the muscle mass of patients with those GSDs that are associated with impairments in muscle glycogen breakdown. In any case, it must be noted that increases in muscle strength induced by training can occur with or without concomitant increases in muscle mass (Reggiani & Schiaffino, 2020). Among the possible explanations for the lack of a robust correlation between increases in muscle mass, on the one hand, and gains in muscle strength, on the other, is the contribution of neural adaptations (Reggiani & Schiaffino, 2020), because training implies learning of the relevant motor unit recruitment and firing rate, together with simultaneous de‐activation of antagonist muscles (Mason et al., 2019; Moritani & deVries, 1979). Some protocols of resistance training might not be suitable to develop neural control of the hypertrophic muscles sufficiently, whereas some other protocols (such as ours) might be sufficient to improve neural motor control but not to stimulate muscle hypertrophy. In this effect, owing to the risk of muscle pain and rhabdomyolysis associated with exercise in the context of many GSDs [such as GSD‐V (Lucia et al., 2021), but also GSDIII (Hennis et al., 2022; Preisler et al., 2013)], resistance exercise sessions (especially if not supervised in person) should probably not be focused on gaining muscle mass.

Several limitations of the present study should be acknowledged, notably the small sample size, which precluded the use of a controlled design. GSD‐IIIa is a rare condition, hindering the recruitment of large cohorts of participants. In fact, we contacted all diagnosed Spanish patients with GSD‐IIIa and enrolled the majority in our study.

There are several strengths and novelties in our work, including not only assessing the effects of an exercise intervention in this patient population, but also the integration of numerous measurements at the multisystem level.

## 5 CONCLUSION

A combined, remotely supervised home‐based 12 week endurance and strength training intervention seems to be tolerable and feasible in patients with GSD‐IIIa, exerting numerous beneficial effects, including a reduced perception of fatigue and decreased VAT levels, an improved cardiorespiratory fitness and lower‐body muscle strength, and healthier cardiac remodelling. These findings might be important from both clinical and physiological perspectives, providing support for the potential beneficial effects of physical exercise at the multisystem level.

## AUTHOR CONTRIBUTIONS

Asunción Bustos‐Sellers: study design, manuscript editing, training supervision and data collection (all study outcomes except echocardiography and blood biomarkers). Almudena Montalvo‐Pérez: training supervision and data collection (all study outcomes except echocardiography and blood biomarkers). Araceli Boraita: data collection (echocardiography). Gonzalo Saco‐Ledo: data collection (diet questionnaires). Lidia B. Alejo, David Barranco‐Gil, Pedro L. Valenzuela, Tomàs Pinós: data collection (cardiopulmonary exercise and/or strength testing). Nerea López‐Maldonado: patient recruitment and clinical management, clinical data collection and study design. Roser Ferrer‐Costa, Javier Esparcia‐Estrela, Celia Monteagudo‐López: data collection (blood biomarkers). Alejando Santos‐Lozano: data collection (assistance in echocardiographic analyses) and statistical analyses. Alfredo Santalla and Alejandro Lucia: study design, manuscript editing, data collection (cardiopulmonary exercise testing). All authors reviewed the manuscript in depth for intellectual content. All authors approved the final version of the manuscript and agree to be accountable for all aspects of the work in ensuring that questions related to the accuracy or integrity of any part of the work are appropriately investigated and resolved. All persons designated as authors qualify for authorship, and all those who qualify for authorship are listed.

## CONFLICT OF INTEREST

None declared.

## Supporting information



Supplementary Materials.

**Supplemental File 1**. Four‐day dietary intake [using the Automated Self‐Administered 24‐h (ASA24©) Dietary Assessment Tool].
**Supplemental File 2**. Blood response to exercise tests.

## Data Availability

Individual data are included as a supplementary file.
