# Supplementary material for: Physical exercise intervention in glycogen storage disease IIIa: Feasibility and multisystem benefits
Source: Exp Physiol. 2025 May 31;111(1):153–66. doi: 10.1113/EP092644 (PMC12756873; doi:10.1113/EP092644)
Supplement: Supplementary file 2 — Supplemental File 1. Four‐day dietary intake [using the Automated Self‐Administered 24‐h (ASA24©) Dietary Assessment Tool]. Supplemental File 2. Blood response to exercise tests. [file EPH-111-153-s001.docx]

**Supplemental file 1.** Four-day dietary intake (using the Automated Self-Administered 24-hour (ASA24©) Dietary Assessment Tool).

|  | Baseline | Postintervention | P-value  (effect size*) |
| --- | --- | --- | --- |
| Adults | | | |
| Carbohydrates (%) | 29.4 (24.5-36.0) | 30.0 (26.8-37.9) | 0.144 (0.731) |
| Fats (%) | 20.2 (18.8-23.6) | 16.5 (12.3-20.0) | 0.273 (0.548) |
| Proteins (%) | 50.4 (42.3-55.8) | 54.8 (41.6-62.6) | 0.715 (0.183) |
| Added Sugars (g) | 18.6 (14.9-21.1) | 27.1 (18.7-32.1) | 0.273 (0.548) |
| Saturated fats (g) | 31.5 (22.0-40.0) | 17.3 (15.4-22.4) | 0.285 (0.535) |
| Carbohydrates (g) | 136.8 (119.1-151.4) | 123.6 (103.4-157.3) | 0.715 (0.183) |
| Proteins (g) | 126.0 (114.4-137.1) | 113.6 (92.6-139.4) | 1.00 (0.000) |
| Proteins (g/kg) | 3.1 (2.6-3.4) | 3.2 (2.5-3.4) | 0.593 (0.268) |
| Fats (g/kg) | 1.4 (1.1-1.6) | 0.8 (0.7-11) | 0.357 (0.461) |
| Carbohydrates (g/kg) | 2.0 (1.7-2.1) | 1.4 (1.3-1.8) | 0.465 (0.365) |
| Children | | | |
| Carbohydrates (%) | 27.0 (25.8-31.6) | 23.8 (22.9-27.5) | 0.138 (0.663) |
| Fats (%) | 19.7 (19.4-22.6) | 16.9 (15.3-19.2) | **0.043** (0.905) |
| Proteins (%) | 48.7 (47.5-53.6) | 58.9 (52.6-60.1) | **0.043** (0.905) |
| Added Sugars (g) | 17.8 (14.3-28.0) | 12.0 (5.5-14.3) | 1.000 (0.000) |
| Saturated fats (g) | 33.3 (30.0-58.5) | 26.5 (25.0-46.3) | 0.068 (0.817) |
| Carbohydrates (g) | 156.3 (139.8-162.3) | 124.3 (97.3-139.0) | 0.068 (0.817) |
| Proteins (g) | 161.0 (150.0-186.3) | 167.8 (150.0-192.8) | 0.715 (0.163) |
| Proteins (g/kg) | 6.8 (6.4-7.4) | 6.8 (5.7-7.6) | 0.465 (0.327) |
| Fat (g/kg) | 2.7 (2.4-3.8) | 3.0 (2.1-3.0) | 0.174 (0.609) |
| Carbohydrates (g/kg) | 3.7 (3.1-3.7) | 3.0 (2.2-3.1) | 0.068 (0.817) |

Data are median (interquartile range) and the sample size was n= 4 (adults) and n=5 (children) for all outcomes. Significant p-values are in bold. Symbol: * the effect size was calculated using the rank-biserial correlation.

**Supplemental file 2.** Blood response to exercise tests.

|  | Baseline | Postintervention | P-value  (effect size*) |
| --- | --- | --- | --- |
| Pre-exercise |  |  |  |
| Glucose (mg/dL) | 99 (89-109) | 102 (90-110) | 0.624 (0.164) |
| Lactate (mM) | 1.2 (0.9-1.6) | 1.0 (0.9-1.3) | 0.953 (0.197) |
| Ketones (mM) | 0.1 (0.0-0.2) | 0.2 (0.1-0.2) | 0.624 (0.400) |
|  |  |  |  |
| Constant load test |  |  |  |
| Glucose (mg/dL) | 97 (93-114) | 94 (90-110) | 0.314 (0.336) |
| Lactate (mM) | 1.4 (0.9-1.7) | 1.1 (0.9-1.2) | 0.342 (0.316) |
| Ketones (mM) | 0.1 (0.0-0.2) | 0.1 (0.1-0.2) | 0.336 (0.317) |
|  |  |  |  |
| Ramp test |  |  |  |
| Glucose (mg/dL) | 85 (80-103) | 80 (74-84) | **0.021** (0.770) |
| Lactate (mM) | 2.1 (1.3-2.7) | 1.6 (1.3-2.2) | 0.813 (0.790) |
| Ketones (mM) | 0.1 (0.0-0.1) | 0.1 (0.1-0.2) | 0.143 (0.489) |

Data are median (interquartile range) and the sample size was n=9 for all outcomes. The significant p-value is in bold. Symbol: * the effect size was calculated using the rank-biserial correlation.
